# Neuromotor control associates with muscle weakness observed with McArdle sign of multiple sclerosis

**DOI:** 10.1002/acn3.51526

**Published:** 2022-03-15

**Authors:** Nathan D. Schilaty, Filippo Savoldi, Zahra Nasr, Brian G. Weinshenker

**Affiliations:** ^1^ Department of Neurosurgery & Brain Repair University of South Florida Tampa Florida USA; ^2^ Center for Neuromusculoskeletal Research University of South Florida Tampa Florida USA; ^3^ Department of Neurology Mayo Clinic Rochester Minnesota USA

## Abstract

**Objective:**

Multiple Sclerosis (MS) is often accompanied by myelopathy, which may be associated with progressive worsening. A specific finding of MS‐associated myelopathy is McArdle sign, wherein neck flexion is associated with prominent increased limb weakness relative to that detected with neck extension. In this study, we characterized neuromotor control properties of finger extensors in association with the McArdle sign.

**Methods:**

A custom‐built device was utilized to monitor torque production of the wrist extensors with simultaneous recording of surface electromyography of the extensor digitorum. The electromyography was decomposed and analyzed via both linear and nominal regressions.

**Results:**

Linear regressions demonstrated a strong difference between groups for MS from healthy controls and other myelopathies for motor unit action potential amplitude and average firing rate (*p* < 0.001). Further, linear regression demonstrated good correlations of neuromotor variables to mechanical torque output (0.24 ≤ *R* ^2^ ≤ 0.76). Nominal regression distinguished MS from healthy controls with an AUC of 0.87, specificity of 0.97, and sensitivity of 0.64. Nominal regression of MS from other myelopathies demonstrated an AUC of 0.88, specificity of 0.85, and sensitivity of 0.79.

**Interpretation:**

These data demonstrate the neuromotor control factors that largely determine muscle force production change with the observation of McArdle sign; these neuromotor control factors can differentiate MS from both healthy controls and other myelopathy conditions.

## Introduction

In 1987, O'Neill et al. described a clinical sign in a patient with multiple sclerosis (MS), who developed difficulty walking during neck flexion and walked with their neck hyperextended. The eponym “McArdle Sign” (McS) recognizes M.J. McArdle for teaching about this sign in clinical practice.^1^, ^2^ Until recently, this sign has not been further studied to determine its specificity or clinical utility for diagnosis of MS.^3^, ^4^

We recently conducted a blinded study to assess the frequency and specificity of this sign for MS. The strength of the extensor digitorum, a sensitive and convenient muscle in which the McS can be detected, was quantitated with a torque measurement device to detect changes that occur with neck flexion compared to extension.^3^ McS was additionally detectable in other muscle groups sensitive to upper motor neuron lesions. The blinded cross‐sectional study demonstrated that a 10% reduction in strength with flexion was 100% specific and 63% sensitive using a receiver operator curve (ROC) analysis comparing patients with a variety of other non‐MS myelopathies.^3^ O'Neill et al., who reported this finding in a single patient, did not recognize that McS was common.^1^ This sign is usually not associated with clinical symptoms and marked impairments of neck flexion clinically manifest only in patients with moderate MS presentation. Nonetheless, detection of the sign by clinical examination can be a helpful way of supporting a diagnosis of demyelination in patients with myelopathy of uncertain cause. Another sub‐study from this cohort demonstrated that MS patients had decreased muscle stiffness and increased neuromechanical error with the neck in flexion.^4^ We found a strong correlation between clinically and instrument‐based measurements, providing opportunity to objectively quantify McS.^3^, ^4^

Our prior studies concentrated on peak strength and biomechanical stiffness. Here, we evaluated the neuromotor control of McS by assessment of decomposed electromyography (dEMG)^5^, ^6^, ^7^, ^8^ of the extensor digitorum musculature as a result of head position (i.e., neck flexion/extension) to determine the neural contribution of individual motor units (MUs) to the McS phenomenon. The objective of this study was to demonstrate an association of neuromotor control strategies with biomechanical changes of McS as disorganization of normal MU firing patterns can compromise MU force generation.^9^

Voluntary force production is achieved by neural drive to muscle, which can be assessed EMG techniques; EMG measures electrical activity of muscles––a stochastic signal from observed motor unit action potentials (MUAPs). Previous work has shown that muscle force generation is proportional to both rate coding (pulses per second; pps) and quantity of motor unit (MU) recruitment.^10^ Thus, to achieve additional force, neural activity adapts with a combination of increased rate coding and/or MU recruitment. Previous work has utilized dEMG to investigate the relationship between rate coding and recruitment threshold during voluntary isometric contractions.^7^, ^11^ In healthy subjects, MU recruitment threshold contributed to MU rate coding characteristics, depicted by an inverse linear relationship.^7^ Normal MU characteristics and variable effects of interventions on MU characteristics have been reported.^11^, ^12^, ^13^, ^14^, ^15^ However, MU characteristics and neuromotor control strategies associated with disease states such as MS have not been well characterized.

With this substudy, we hypothesized that dEMG would demonstrate increased MU size, increased recruitment thresholds, and decreased firing rate (FR) for MS patients with the neck in flexion compared to other groups, indicative of neural inhibition and mechanically induced conduction block. We further hypothesized that MS would demonstrate differences in these dEMG metrics between neck extension and flexion––neuromotor inhibition that causes muscle weakness observed with McS (decreased isoinertial strength).^3^ This knowledge about neuromotor control adaptations during neck flexion/extension may provide further insight of the mechanism of McS and adaptive neuromotor strategies of MS.

## Methods

The research protocol was carried out in accordance with the Declaration of Helsinki and was approved by Mayo Clinic's internal review board (#15–009112). All patients signed an informed consent and privacy of human subjects was maintained. From the cohort of 106 patients evaluated in the parent study, 48 participants participated in this substudy [age: 49 years (13)]. Two subjects did not have interpretable data and were excluded. Of the 46 patients, 17 were healthy controls (CTRL); of those with finger extensor weakness, 16 were patients diagnosed with MS, and 13 with other myelopathies (OM; Table 1). OM were diverse and included compressive myelopathy, amyotrophic lateral sclerosis, intrinsic spinal cord tumor, neuromyelitis optica, neurosarcoidosis, and other myelopathies. Patients were not selected based on presence of McS; the only requirement was some degree of detectable finger extensor weakness, even if mild. The OM group was comprised of myelopathy patients of different etiologies. Enrollment was monitored by diagnosis to ensure that the desired number of patients in each diagnostic category was included. The study population was a convenience sample, although we attempted to enroll consecutive patients who met study criteria.

**Table 1:** Clinical and demographic characteristics by group.

	MS (*n* = 16)	OM (*n* = 13)	CTRL (*n* = 17)	*p*‐value
Age				
Mean years (SD)	54 (11)	52 (15)	43 (12)	0.033^a^
Side tested				
Left	6	8	7	0.389^b^
Right	10	5	10	
Sex				
Male	5	7	8	0.442^b^
Female	11	6	9	
EDSS score [range 0–10]				
Median score (25th, 75th)	4 (3, 6)	3 (2, 6)	NA	0.173^c^
Disease duration				
Median years (25th, 75th)	17 (6, 23)	2 (1, 3)	NA	<0.001^c^
Cervical spine MRI				
Quantity	16	11		0.429^b^
Time from test, months (25th, 75th)	1 (0, 11)	1 (0, 4)	NA	0.834^c^
Location of T2‐weighted cervical cord lesion to weakness				
None	0	2	NA	0.262^b^
Ipsilateral	2	1		
Contralateral	0	0		
Bilateral	14	8		
Atrophy in cervical spine				
None	15	8	NA	0.295^b^
Ipsilateral	1	1		
Contralateral	0	1		
Bilateral	0	1		
MS phenotype				
Clinically isolated syndrome (CIS)	0	NA	NA	NA
Relapsing–remitting MS (RRMS)	7			
Primary progressive MS (PPMS)	4			
Secondary progressive MS (SPMS)	5			
Types of other myelopathies				
Compressive myelopathy	NA	2	NA	NA
Amyotrophic lateral sclerosis		2		
Intrinsic spinal cord tumor		1		
Neuromyelitis optica		2		
Neurosarcoidosis		2		
Other myelopathy		4		

Statistical tests: ^a^Analysis of Variance; ^b^Fisher Exact; ^c^Wilcoxon/Kruskal–Wallis.

EDSS, expanded disability status scale [0 = normal to 10 = death]; MS, Multiple Sclerosis; OM, other myelopathies.

### Testing protocol

**Figure 1:**
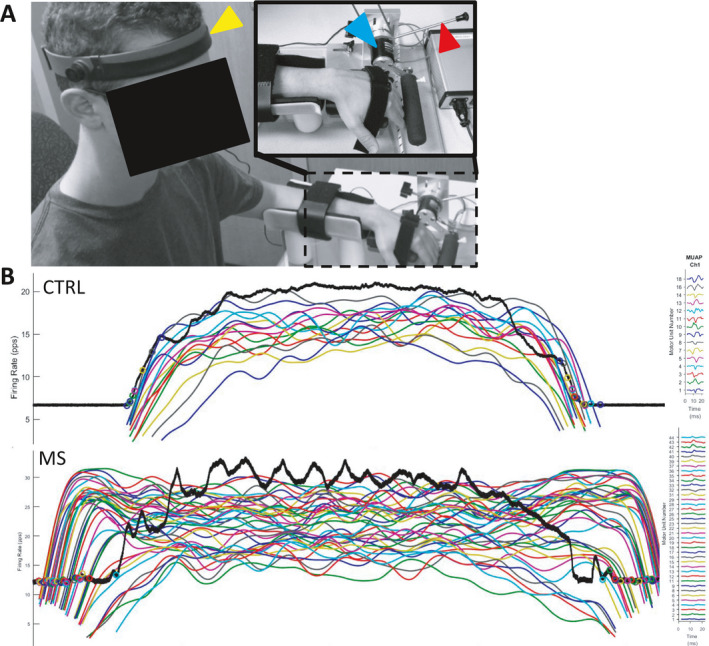
McArdle test setup and sample decomposed electromyography. (A) The arm was secured in a custom‐built device for measurement of finger extensor torque production. The hand was positioned so that the axes of rotation of the metacarpophalangeal joints were in‐line with the rotation axis of the torque cell. The torque cell (blue arrow) measured torque as the subject pushed against a padded comfort bar. Two gyroscopes in the headband measured neck flexion/extension (yellow arrow). The lever was utilized by the examiner to apply a downward force that the subject was asked to resist (red arrow). The EMG electrodes were place on the muscle belly of the extensor digitorum of the arm in the McArdle device. (Adapted from Schilaty et al. Biomechanical muscle stiffness measures of extensor digitorum explain potential mechanism of McArdle Sign. *Clin Biomech*. 2021; 82:105277). (B) Sample dEMG data demonstrates characteristic differences observed between subjects. The black line represents force production (30% MVIC target). The colored lines represent recruitment of a particular motor unit, average firing rate, and derecruitment of the motor unit. Comparison of the two signals may suggest “MU disorganization” of one group compared to another. [Colour figure can be viewed at wileyonlinelibrary.com]

A custom‐built finger extension device secured the forearm and hand with Velcro straps and a torque cell (RTS‐500, Transducer Techniques; Temecula, CA) was placed in‐line with the axis of the metacarpophalangeal joints with an attached comfort bar (Fig. 1A). The bar was positioned above the fingers in near full extension so that immediate effort of finger extension registered torque production, similar to clinical performance of McS with a manual strength test. A technician, blinded to clinical diagnosis, performed the McS evaluation either: (1) clinically (by hand), and (2) with the device in which downward pressure was placed on the lever that depressed the bar positioned above the fingers. The discrete McS score was recorded (grade 0–3; 0 = no weakness; 3 = marked weakness).

Patients were fitted with a two‐axis gyroscopic headband to measure accurate flexion/extension position of the neck (Fig. 1A). All gyroscopic and torque cell data were collected at 1 kHz via proprietary *LabVIEW* programmed software (v2016; National Instruments, Austin, TX).

After preparation of the electrode site with dry shaving and isopropyl alcohol, a 5‐pin dEMG electrode (Delsys; Natick, MA) was affixed over the belly of the extensor digitorum according to SENIAM standards.^16^ A signal quality check was performed to ensure EMG noise level was <20 *μ*V. Two paradigms of muscle contraction were utilized: Isoinertial (patient extended fingers against constant resistance of continuous downward movement of the bar at a constant rate by lab technician) and isometric. As the dEMG technology was not capable to track motor units during dynamic contractions, isometric contractions were utilized (Fig. 1B). For the isoinertial paradigm, five paired successive trials of neck extension followed by neck flexion were performed. The first set of measurements in extension/flexion were discarded to mitigate learning effects. Peak torque values for each trial were extracted from raw data using MATLAB software (MathWorks, Inc.; v2016a). Reduction in torque between the paired trials in neck extension and flexion position was calculated as follows: [(Torque_ext_ − Torque_flex_)/Torque_ext_*100]. This value was averaged over the last four trials (percent difference IsoTorque). For the isometric paradigm, the patients first performed three maximal voluntary isometric contractions (MVIC) of finger extension. Patients then performed three trials of isometric contractions with the neck randomized across: neutral, flexion, or extension.^3^ As the neutral position did not demonstrate any statistical difference from extension, the two were combined for analysis. Each isometric contraction followed a trapezoidal waveform with a 3‐sec ramp up, 10‐sec steady phase (30% MVIC), and a 3‐sec ramp down. Rest of 40 sec was allotted between each isoinertial/isometric trial. All EMG data were oversampled at 20 kHz to avoid introduction of significant phase skew across channels.

### EMG signal decomposition

EMG decomposition was performed with *dEMG Analysis* software (Delsys; Natick, MA). The analog EMG channel data were band‐pass filtered (20–1750 Hz). The digital EMG signals were digitally filtered using a high‐pass filter with a cutoff frequency of 50 Hz prior to decomposition.^6^ The signal decomposition algorithm first extracted action potential “templates” of as many MUAPs as possible from the input EMG signal. The algorithm then searched for signal regions where the extracted MUAP templates were superimposed with other identified MUAPs or with unidentified action potentials. The algorithm takes both constructive and destructive interference effects into account when analyzing such superpositions. Moreover, the algorithm requires that the unidentified action potentials account for less than 25% of the signal energy at the firing locations of the decomposed MUAPs.^6^

To verify the decomposed signal, the algorithm performed a Decompose‐Synthesize‐Decompose‐Compare test.^17^ The original signal was decomposed, as described in the preceding paragraph. Next, white noise with an RMSE value equivalent to the residual of the non‐decomposed signal was added to the decomposed signal and synthesized. The synthesized signal was then decomposed, as described above, and compared to the original signal decomposition. Only MUs with an accuracy of ≥90% were included in analysis for the current study. In addition to internal validation by the development group, the decomposition algorithm has been externally validated against spike triggered averaging techniques^6^, ^18^ and has been validated for high reliability to needle electromyography.^6^, ^7^, ^8^

Common drive was calculated by low‐pass filtration of the FRs of each individual MU through a Hanning window and subsequently detrending using a high‐pass filter (0.75 Hz). The detrended MU FRs were then cross‐correlated and values of amplitude and latency extracted. Additional detail on the methodology is available at.^19^, ^20^, ^21^, ^22^

Delta *F* (Δ*F*) was evaluated with custom *LabVIEW* software (v2020; National Instruments, Austin, TX) according to descriptions in the literature.^23^, ^24^, ^25^, ^26^ Briefly, Δ*F* values were calculated for every possible pair of MUs in each contraction with a ‘control MU’ and all other MUs treated as a “test MU.” The instantaneous FRs of both MUs were smoothed by a fifth‐order polynomial fit and the Δ*F* values were calculated from the polynomials by taking the difference between the values of the control MU at recruitment and derecruitment of the test unit. Δ*F* is a paired MU analysis and an indirect technique for estimating synaptic activity due to the magnitude of persistent inward currents in human motor neurons.^24^ For Δ*F* analysis, MUs were only included that achieved ≥0.5 *R* ^2^ values with the referent MU and ≥0.5 impulses/sec.^23^, ^24^, ^27^ Although previous literature utilized a cutoff *R* ^2^ ≥ 0.7,^23^, ^24^ we determined to include ≥0.5 to maintain integrity of more representative MUs in the analyses (68.9%) and allow for additional generalizability across MUs.

### Statistical analysis

Outcome variables of interest for this study included MUAP peak‐to‐peak amplitude (MUAP‐P2P), MU average (AvgFR), initial (InitFR), and terminal FR (TermFR), MU recruitment threshold, neural drive (AvgFR–InitFR), isoinertial difference between neck extension and flexion (Ext–Flex),^3^, ^4^ and common drive coefficient and latency (cross‐correlation of MUs). Data were imported into *JMP 14 Pro* (SAS Institute Inc., Cary, NC) for statistical analysis. Demographics were assessed with ANOVA, Fisher's Exact, Wilcoxon, or Kruskal–Wallis tests based on the normality of the data. Standard least squares linear regressions were performed with continuous variables of interest with associated least squares means ANOVA/*t*‐test and Tukey's post hoc comparisons for ANOVAs. A Self‐Organizing Map cluster analysis was performed on the key variables that differentiated groups (AvgFR, MUAP‐P2P, and Δ*F*). Linear regressions were performed with all possible factor combinations (full factorial) of AvgFR, MUAP‐P2P, and Δ*F* to demonstrate the neuromotor control association with mechanical output. Similarly, nominal regressions were performed with a full factorial of AvgFR, MUAP‐P2P, Δ*F*, and percent difference IsoTorque to determine their ability to predict diagnostic group from neuromotor control. As an assumption for linear regression is normality of data, nonparametric data were transformed as appropriate (Cube root: Recruitment Threshold, Derecruitment Threshold; Log: MUAP‐P2P). Statistical significance was set a priori at *α* < 0.05.

## Results

Clinical and demographic characteristics are summarized in Table 1.

Decomposition identified 4393 MUs, of which 1543 were from CTRLs, 1668 from MS, and 1182 from OM. For each group, the median number of MUs identified from the decomposition algorithms was similar (*χ* ^2^ = 3.151; *p* = 0.207) with 11^6^, ^17^ for CTRLs, 11^5^, ^17^ for MS, and 11^5^, ^18^ for OM. One‐dimensional MU characteristics stratified by measurements with neck extension and flexion are summarized in Table 2.

**Table 2:** Motor unit firing rates (FR) as assessed by one‐dimensional statistical analyses (*t*‐test or Wilcoxon) between neck extension and neck flexion.

	Neck position		*p*‐value (between neck position)	*p‐value* (between groups)
	Extension	Flexion		
InitFR (Hz) (mean, SD)				
CTRL	6.5 (2.1)	6.5 (1.9)	0.758	**<0.001**
MS	5.9 (2.0)	6.3 (2.5)	**<0.001**	
OM	5.2 (1.9)	5.2 (1.9)	0.556	
AvgFR (Hz) (mean, SD)				
CTRL	18.0 (3.7)	17.7 (3.3)	0.131	**<0.001**
MS	16.3 (3.6)	16.2 (4.3)	0.857	
OM	15.5 (4.6)	15.5 (4.4)	0.917	
TermFR (Hz) (mean, SD)				
CTRL	8.2 (2.7)	7.9 (2.4)	**0.006**	**<0.001**
MS	7.7 (2.7)	7.8 (3.3)	0.655	
OM	7.1 (2.5)	6.8 (2.2)	**0.011**	
# MU firings (median; 25th/75th quartile)				
CTRL	255 [205, 306]	243 [205, 291]	0.061	**<0.001**
MS	231 [179, 282]	221 [168, 280]	0.186	
OM	208 [153, 264]	204 [154, 252]	0.266	
Recruitment threshold (median; 25th/75th quartile)				
CTRL	1.5 [0, 8.3]	1.4 [0, 7.7]	0.844	**<0.021**
MS	2.4 [0.3, 9.1]	2.1 [0.3, 10.0]	0.911	
OM	2.3 [0.3, 11.1]	3.2 [0.3, 11.5]]	0.886	

Each *p*‐value reports difference between neck extension and flexion for each group. The most notable differences occur for MS with initial FR and for CTRL and OM for terminal FR. Values in BOLD are significant (*p* < 0.05).

### Motor unit action potentials

**Figure 2:**
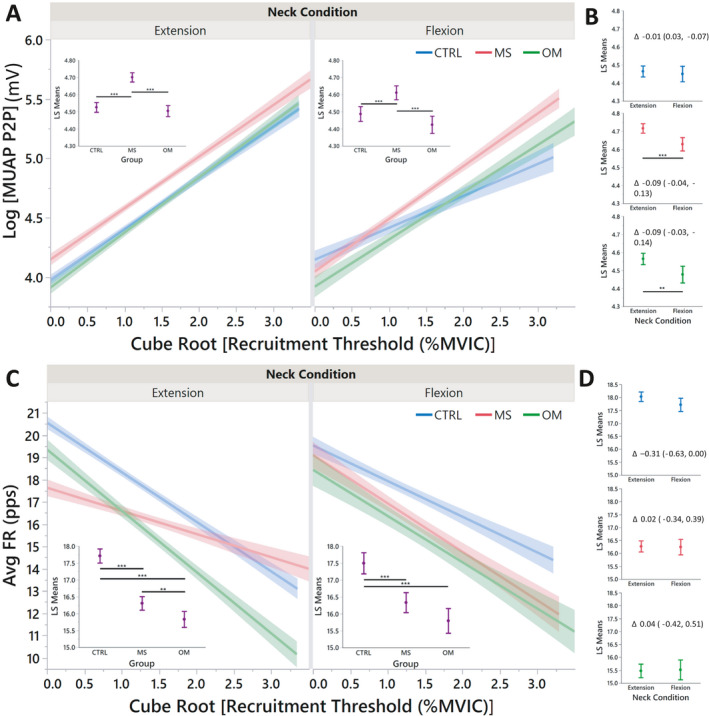
Linear regressions of motor unit action potentials (MUAP) and average firing rate (AvgFR) by recruitment threshold. Each regression (A and C) demonstrates the differences of neuromotor control between groups. *Insets:* Least squares means that summarize each linear regression. Least squares means comparisons (B and D) are demonstrated for each neck condition between groups with a delta value and 95% confidence intervals. *P*‐values are demonstrated by *, **, and *** for <0.05, <0.01, and <0.001, respectively. [Colour figure can be viewed at wileyonlinelibrary.com]

Standard least squares linear regression (*R* ^2^ = 0.46 extension; *R* ^2^ = 0.34 for flexion) revealed an increased MUAP in patients with MS across all levels of recruitment in both neck flexion and neck extension compared to CTRL and OM (*p* < 0.001; Fig. 2A). A further comparison within groups between neck extension and flexion (Fig. 2B) revealed no differences for CTRL (*p* = 0.594), but lower MUAP with neck flexion compared to neck extension for MS (*p* < 0.001) and OM (*p* = 0.003).

### Motor unit firing rates

Standard least squares linear regression (*R* ^2^ = 0.30 extension; *R* ^2^ = 0.24 for flexion) revealed lower AvgFR for both MS and OM compared to CTRL in neck extension (*p* < 0.001) and neck flexion (*p* < 0.001; Fig. 2C). Further, OM had lower AvgFR than MS in neck extension (*p* = 0.008), but not in neck flexion (*p* = 0.064). A further comparison within groups between neck extension and flexion (Fig. 2D) revealed no differences for CTRL (*p* = 0.051), MS (*p* = 0.901), and OM (*p* = 0.862).

### Δ*F*

ANOVA of Δ*F* with neck extension demonstrated a mean value of CTRL at −1.59. MS was 1.37 greater than CTRL (*p* < 0.001) and OM was 0.11 lower than CTRL (*p* = 0.816). MS was 1.48 higher than OM (*p* < 0.001). With the neck in flexion, ANOVA of Δ*F* slightly changed for CTRL with a mean of −0.53. Neither MS nor OM differed from CTRL in neck flexion (*p* = 0.430, *p* = 0.792, respectively) and MS and OM did not differ (*p* = 0.191).

### Common drive coefficient and latency

The common drive coefficient was summarized (median) within all identified MUs of each individual. Kruskal–Wallis analysis of common drive revealed no differences between groups with neck extension (common drive coefficients were 0.33 [0.32, 0.35], 0.33 [0.31, 0.35], and 0.35 [0.33, 0.39] for CTRL, MS, and OM, respectively; *p* = 0.141); however, with neck flexion the group differences differed (common drive coefficients 0.32 [0.31, 0.36], 0.34 [0.34, 0.4], and 0.36 [0.31, 0.49] for CTRL, MS, and OM, respectively; *p* = 0.027). Post hoc comparison revealed MS greater than CTRL (*p* = 0.012) and OM greater than CTRL (*p* = 0.045). A paired subtraction (Ext‐Flex) within individuals (*p* = 0.059) identified 0.01 [−0.02, 0.03] for CTRL, −0.03 [−0.06, 0.0] for MS, and 0.0 [−0.08, 0.03] for OM. Post hoc comparison demonstrated MS less than CTRL (*p* = 0.021). There were no differences of common drive latency between neck conditions or between groups.

### Association of neuromotor control variables with torque output

**Figure 3:**
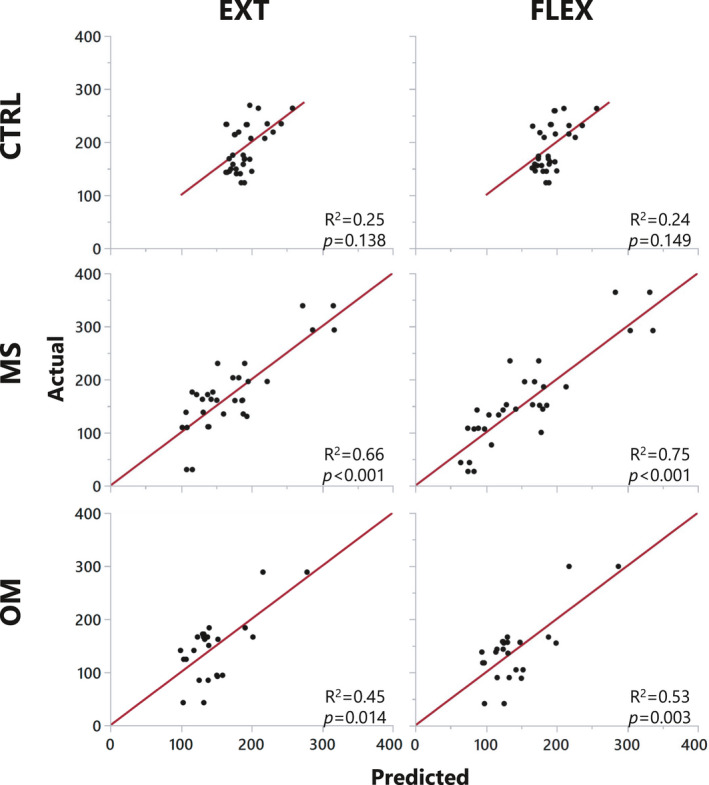
Linear regressions of isoinertial torque versus neuromotor control strategies. A full factorial of the variables AvgFR, MUAP‐P2P, and Δ*F* were utilized to predict isoinertial torque. For both neck conditions, all groups had an *R* ^2^ ≥ 0.24. This data demonstrates the high correlation of three physiological neuromotor control variables (and their interactions) to the development of isoinertial torque strength. (Note: the x‐ and y‐axes are units of ounce‐inches). [Colour figure can be viewed at wileyonlinelibrary.com]

All MUs were summarized (median) for each individual for both flexion and extension for the neuromotor control variables AvgFR, MUAP‐P2P, and Δ*F*. A full factorial of these variables were utilized in a linear regression model (Fig. 3). Isoinertial torque could be predicted for each group, most clearly with MS. CTRL had an *R* ^2^ = 0.25 and *R* ^2^ = 0.24, MS *R* ^2^ = 0.66 and *R* ^2^ = 0.75, and OM *R* ^2^ = 0.45 and *R* ^2^ = 0.53 for extension and flexion, respectively.

**Figure 4:**
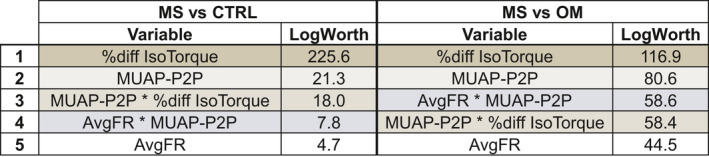
Independent variables for prediction of multiple sclerosis from healthy controls (CTRL) or other myelopathies (OM). Variables are listed with their respective LogWorth value contribution to the resultant nominal regression model. The five remaining variables (color‐coded) are the same for both groups, but with different weighting and order. [Colour figure can be viewed at wileyonlinelibrary.com]

**Figure 5:**
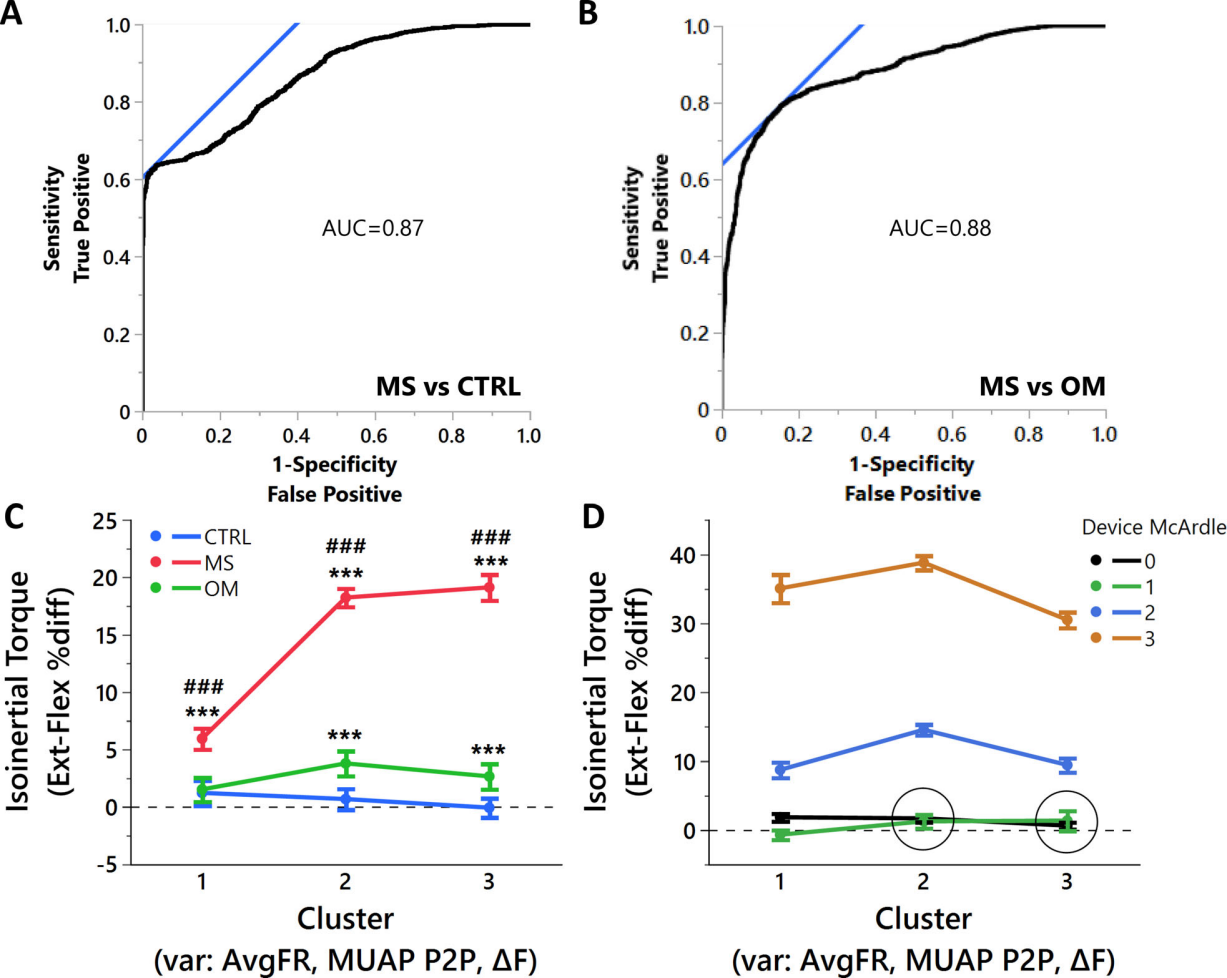
Interrelationship of isoinertial torque with neuromotor control variables. For the nominal regressions (A and B), the variables of AvgFR, MUAP‐P2P, Δ*F*, and percent difference IsoTorque were provided with a full factorial for interactions and reduced to five variables (see Figure 4). (A) MS can be distinguished from CTRL with a specificity of 0.97 and sensitivity of 0.64. (B) MS can be distinguished from CTRL with a specificity of 0.85 and sensitivity of 0.79. The variables of interest included in the cluster analysis were AvgFR, MUAP‐P2P, and Δ*F*. (C) Cluster determination of percent difference IsoTorque by Group. *** designates significance *p* < 0.001 from CTRL and ### demonstrate significance *p* < 0.001 from OM. (D) Cluster determination of %diff of IsoTorque by McArdle grade. All McArdle grades are *p* < 0.001 from one another except within circles. [Colour figure can be viewed at wileyonlinelibrary.com]

A nominal regression with a full factorial of the variables AvgFR, MUAP‐P2P, Δ*F*, and percent difference IsoTorque resulted in a model of 15 independent variables; to avoid model overfit, less significant variables were removed until five independent variables remained. The final model contained (1) percent difference IsoTorque, (2) MUAP‐P2P, (3) MUAP‐P2P * percent difference IsoTorque, (4) AvgFR * MUAP‐P2P, and (5) AvgFR * percent difference IsoTorque (Fig. 4). The model (*χ* ^2^ = 1554.1; *p* < 0.001) had an AUC of 0.87, and identified MS from CTRL with a specificity of 0.97 and a sensitivity of 0.64 (Fig. 5A). A similar model was performed for MS versus OM. The final model contained (1) percent difference IsoTorque, (2) MUAP‐P2P, (3) AvgFR * MUAP‐P2P, 4) MUAP‐P2P * percent difference IsoTorque, and (5) AvgFR (Fig. 4). The model (*χ* ^2^ = 1389.6; *p* < 0.001) had an AUC of 0.88, and identified MS from OM with a specificity of 0.85 and a sensitivity of 0.79 (Fig. 5B).

A Self‐Operating Map cluster analysis resulted in three clusters and an optimal Cubic Cluster Criterion of −31.201. The resultant principle components (eigenvalues of 1.61, 1.00, and 0.39) were utilized in a standard least squares regression to predict percent difference IsoTorque. The model (*R* ^2^ = 0.33; *p* < 0.001) demonstrated excellent differentiation of isoinertial torque between groups (Fig. 5C) with MS different from both OM and CTRL for all three clusters (*p* < 0.001) and OM different from CTRL for clusters 2 and 3 (*p* < 0.001). Similarly, the cluster formulas (File S1) were utilized in a standard least squares regression to predict percent difference IsoTorque. The model (*R* ^2^ = 0.67; *p* < 0.001) demonstrated excellent differentiation of isoinertial torque between all McArdle grades for Cluster 1 (*p* < 0.001), and Grades 3 and 2 different from one another and from Grades 0 and 1 for both Cluster 2 and 3 (*p* < 0.001; Fig. 5D).

## Discussion

This study demonstrated neuromotor control at the MU level in relation to McS, a phenomenon specific to individuals with MS characterized by reduced limb strength, typically assessed in finger extensors, with neck flexion compared to extension. We detected group differences between those with MS, OM, and CTRLS in MUAP, AvgFR, Δ*F*, and common drive. Specifically, (1) individuals with MS had larger MUAPs compared to CTRL and OM during both neck flexion and extension (Fig. 2A and B), (2) individuals with MS had lower AvgFR compared to CTRL in both neck flexion and extension (Fig. 2C and D), (3) MS had higher common drive than CTRL in neck flexion, and 4) MS had higher Δ*F* than CTRL and OM with differences of neck extension and flexion. Thus, our hypotheses that dEMG would demonstrate increased size of MUs, increased recruitment thresholds, and decreased FR in neck flexion for MS patients were partially supported. Further, the neuromotor variables AvgFR, MUAP‐P2P, and Δ*F* demonstrated good correlation of torque production. A nominal regression and cluster analysis of these variables differentiated MS from both CTRL and OM (Fig. 5), supporting our hypothesis that individuals with MS manifest neuromotor inhibition, possibly due to stretch‐associated conduction block, that causes muscle weakness observed with McS.

Standard FR statistics (InitFR, AvgFR, TermFR, #MU Firings, and Recruitment Threshold) were evaluated for all groups (Table 2). These MU characteristics demonstrate the ability of dEMG technology to track MU patterns as previously observed.^28^, ^29^ Individuals with MS had higher InitFR with the neck in flexion compared to extension but no differences in TermFR were detected compared to CTRL and OM (Table 2). Typical MU behavior involves recruitment, acceleration, maintenance of firing frequency, and then de‐acceleration and derecruitment at a lower FR than at recruitment (termed hysteresis).^30^ In flexion, MS individuals recruited MUs at a higher initial FR than CTRL and OM in flexion versus extension but showed no difference in TermFR as the other groups did with flexion. All dEMG metrics differed between groups, especially between CTRL from both MS and OM.

Common drive is a measurement of the degree of cross‐correlation between MU FRs.^20^ Common drive is considered a relative simple strategy of the CNS for controlling MUs and maintaining efficiency.^20^ The common drive coefficient is an indicator of CNS neuronal synchrony.^31^, ^32^, ^33^ The common drive observed herein demonstrated no differences between groups with the neck in extension (common drive coefficient interquartile range of 0.31–0.39). With the neck in flexion, the common drive coefficient interquartile range increased (0.31–0.49) for both MS and OM, indicating increased synchrony of the MU pool via the CNS. These values are similar to those previously reported.^34^, ^35^ As both MS and OM experienced increased common drive with neck flexion, it is likely that the failure of strength output common to both groups resulted in signalling the CNS for additional input to sustain the contraction.^20^ With the paired subtraction of median values of neck position (Ext‐Flex) within individuals, MS fell below zero (−0.03), indicating a higher common drive from the CNS.

As MUs are *dynamic* during a full contraction and also the large quantity of MUs identified in this study, one‐dimensional statistics may yield false positive results (e.g., InitFR difference of 0.4 Hz). Thus, MUs from dEMG observations should be evaluated with multivariate or nonlinear statistical models^9^, ^36^ to insure meaningful results. Similarly, this multivariate complex relationship is vital to demonstrate the association of mechanical motor output (strength) as it relates to the physiological neuromotor control schema.

With linear regression analysis, MU characteristics and neuromotor control strategies were assessed across recruitment phases. Later recruited MUs are larger^17^, ^37^, ^38^ (see Fig. 2A and B). MS demonstrated larger MUAPs compared to OM and CTRL across all recruitment, but there were lower values of MUAPs in neck flexion compared to neck extension (Fig. 2B). This was also true for OM but not for CTRL. AvgFR was not different between neck extension and flexion nor were differences between groups apparent. However, linear regression demonstrated that with neck extension, MS heavily relies on larger MUs (later recruitment) which changed the overall slope of the fitted line (Fig. 2C). Taken together, the data suggest little influence of neck position on recruitment of MUs in CTRL, but OM and MS experience disorganized normal MU firing patterns with neck flexion that compromise MU force generation.^9^

Δ*F* is a surrogate method for measuring the effect of persistent inward currents for neuromodulation––an alteration of nerve activity.^23^ Higher Δ*F* values equate to increased monoaminergic excitability at synaptic dendrites.^23^ In this study, we observed MS had higher Δ*F* than other groups with neck extension, but these differences were not apparent with neck flexion. This observation suggests that individuals with MS may enhance motor strength with neck extension by neuromodulation when mechanically induced conduction block is prevented, potentially decreasing central drive required to maintain strength when the neck is flexed.

**Figure 6:**
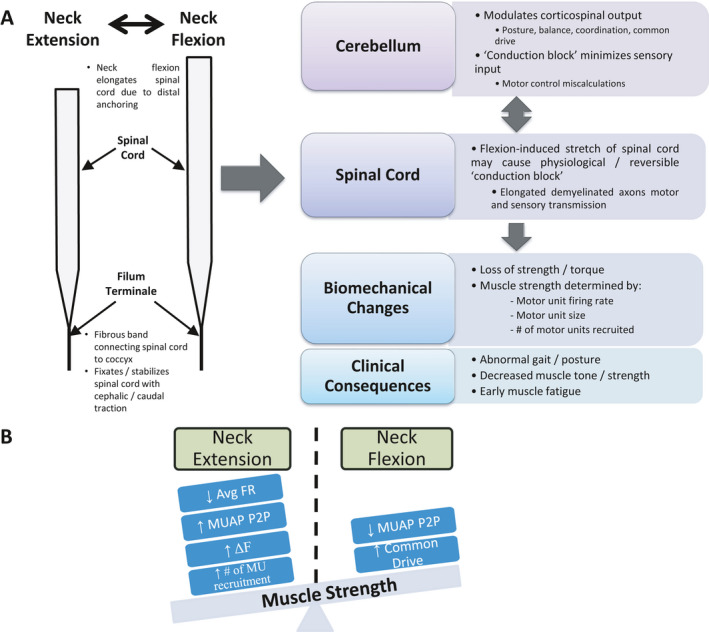
Model of McArdle phenomenon from neuromotor characteristics. (A) As the neck is flexed, the spinal cord elongates. This elongation is suspected to cause “conduction block” in viable demyelinated axons, interfering with modulation of corticospinal output and changes in central common drive to peripheral motor neuron pools. This results in loss of motor strength due to changes in motor neuron firing rate, quantity of motor units recruited, and recruited motor unit size. The clinical consequences from these neuromotor changes result in abnormal gait/posture, decreased muscle tone/strength, and early muscle fatigue. (Image modified from Schilaty et al., 2021) (B) The scale of muscle force/torque is tipped based on these key neuromotor control factors observed. [Colour figure can be viewed at wileyonlinelibrary.com]

We have previously suggested a biomechanical explanation for McS^4^; with the neck in flexion, conduction block caused by elongation of demyelinated axons decreases the efficacy of early recruited small MUs (more electrical resistance) and subsequently utilize larger MUs (less electrical resistance) that would increase EMG intensity.^39^ MS utilized larger MUs than both CTRL and OM across all recruitment. Although MUAP‐P2P decreased in individuals with MS with neck flexion, the MUAP‐P2P regression had a steeper slope than CTRL in neck flexion (Fig. 2A) and the MUAP least‐square mean in flexion was higher for MS compared to CTRL and OM in neck extension (Fig. 2B). Although the MUAPs decreased for MS with neck flexion, AvgFR increased for these MUs associating with evidence of increased common drive during neck flexion. This evidence supports the hypothesis that McS is likely caused by a stretch‐induced CNS conduction.^3^, ^4^ During neck extension, the spinal cord would be less strained (Fig. 6). In this scenario, there are no demonstrable differences of common drive between groups, but MS relies on larger MUs and greater Δ*F*. However, with neck flexion, the spinal cord is strained, and the neurons elongate causing a conduction block of the demyelinated neurons. The result is a decrease of MUs recruited (smaller MUAP‐P2P), increase in common drive as the CNS attempts to respond to lack of MU engagement, and a resultant increase in AvgFR (slope).

Multiple linear regressions demonstrated that a model integrating all combinations of neuromotor control variables AvgFR, MUAP‐P2P, Δ*F* predict isoinertial torque. The linear regressions (Fig. 5) demonstrated good to excellent correlations with highest correlations for MS (*R* ^2^ ≥ 0.66). In fact, the correlations for CTRL would likely be higher with a larger spread of the data, but as CTRLs all clustered on similar values of isoinertial torque, the fit line lost prediction accuracy. These data demonstrate that these neuromotor control factors and their interrelationships are strong predictors of isoinertial torque production measured biomechanically. To further determine contribution of neuromotor control factors of force production, a cluster analysis of AvgFR, MUAP, and Δ*F* highly differentiated MS from other groups with three resultant principal components. Interestingly, the observed clusters demonstrated a profound separation in percent difference of isoinertial torque between neck extension and flexion as well as Grades 2 and 3 of McS (Fig. 5C and D). Thus, a definitive neuromotor control mechanism of MU characteristics exist and clearly demonstrates responsibility for the observed phenomenon of McS.

The differential diagnosis of myelopathy, especially in early stages, is broad. MS is a common cause, but with atypical features, it may be difficult to establish a confident diagnosis. Thus, if neuromotor control characteristics can differentiate various myelopathy presentations, the information can be useful for improving clinical diagnosis beyond customary course of symptoms, response to steroids, lab results, EMG, and radiologic findings. Further, neuromotor control characteristics may be beneficial to monitor treatment progression and observing the effects of prescribed interventions. Nominal regressions with inputs of a full factorial of AvgFR, MUAP‐P2P, Δ*F*, and percent difference IsoTorque were reduced to five primary variables (Fig. 4). Variable reduction greatly simplified the model, avoided overfitting, and showed a near‐identical agreement between group comparisons. The ROCs comparing MS to both CTRL and OM demonstrated near excellent prediction of MS (Fig. 5A and B) with AUCs ≥0.87, specificity ≥0.85, and sensitivity ≥0.64. Combined with the other data published that support this differentiation of MS compared to both CTRL and OM,^3^, ^4^ the data strongly suggests that MS has distinct neuromotor control strategies and resultant biomechanical changes (Fig. 6).

Together, the data presented demonstrates neuromotor control strategies (InitFR, TermFR, Recruitment Threshold, MUAP‐P2P, AvgFR) are altered by MS that contribute to a recently recognized and specific phenomenon, McS. The differences of neuromotor control strategy result in compensatory neuromotor control (Common Drive, Δ*F*). Compared to CTRL, MS demonstrates higher MUAPs, lower AvgFR, decreased neural drive, and increased neuromodulation with neck extension. Patients with OM similarly demonstrated decreased MUAP with neck flexion and overall lower AvgFR. However, neither neural drive nor neuromodulation decreased in individuals with OM with neck flexion and less dramatic differences of cluster analysis compared to CTRL. The differences of MS compared to OM may demonstrate that the stretch‐induced conduction block^4^ may cause sensory neuron miscalculations and prevent appropriate motor output for upregulated neuromodulation and MU acceleration required^30^ to maintain neural drive that is present with neck extension.

Our study has potential limitations. dEMG at low levels of contraction is difficult to measure due to low signal‐to‐noise ratios. Alternatively, low‐threshold MUs may not be well‐represented in the decomposed signal for greater effort‐level trials due to superposition of higher‐threshold MUs. However, the decomposition algorithm considers superposition of MUAP trains. While there has been recent debate about different decomposition techniques, the decomposition algorithm used has demonstrated valid rate coding and MUAP shapes.^6^, ^18^, ^40^ EMG measures electrical activity of the whole muscle, and as previously described, the signal decomposition technique used identifies individual MUAP trains contained in the EMG signal.

Inherent complexities of MU analysis complicate the interpretation of our data. MU behavior is non‐linear in nature^41^; each contraction incorporates multiple MUs that initiate rate coding at independent recruitment thresholds which then accelerate and decelerate in rate coding throughout the demands of the muscle contraction.^42^ Consequently, the relationships between groups can be difficult to parse as a breakdown of singular MU characteristics (i.e., AvgFR, recruitment threshold, etc.) will yield similar characteristics between groups due to averaging (Table 2). Further, with high quantities of MUs identified, the averaged values may be statistically, but not clinically significant; the absolute differences were small (average rate coding 15.9 vs. 16.0). However, when MU characteristics are assessed in a multivariate form^9^ as analyzed herein, a different pattern emerges in which MU strategies become evident in the y‐intercept, slope,^43^ and least‐squares means which adjusts the average based on the multivariate approach. Due to the limited number of data points and only a single value of muscle contraction in this sub‐study, linear regressions were not reported for Δ*F* by recruitment. Future studies can address dEMG for MS across different levels of resistance to finger extension.

## Conclusion

This study is among the first to evaluate neuromotor control characteristics for MS, and specifically utilized this analysis to evaluate a unique‐specific electrophysiologic and clinical phenomenon in MS, McS. We demonstrated differences of neuromotor control strategies of MS compared to both CTRL and OM. Important neural adaptations between neck extension and flexion have been elucidated (increased MUAP, lower AvgFR, increased common drive) that distinguish MS compared to CTRL and OM and demonstrate the neuromechanical integration relevant to McS.

## Conflict of Interest

The authors have no conflict of interest to disclose.

## Supporting information


**File S1.** Cluster Formula Utilized to Predict percent difference IsoTorque. For determination of percent difference IsoTorque, a Self‐Operating Map cluster analysis resulted in three clusters and an optimal Cubic Cluster Criterion of −31.201. The resultant principle components (eigenvalues of 1.61, 1.00, and 0.39) were utilized in a standard least squares regression to predict percent difference IsoTorque. The model (*R* ^2^ = 0.33; *p* < 0.001) demonstrated excellent differentiation of isoinertial torque between groups with MS different from OM and CTRL for all three clusters (*p* < 0.001) and OM different from CTRL for clusters 2 and 3 (*p* < 0.001).
