# Supplementary material for: Neuromotor control associates with muscle weakness observed with McArdle sign of multiple sclerosis
Source: Ann Clin Transl Neurol. 2022 Mar 15;9(4):515–28. doi: 10.1002/acn3.51526 (PMC8994990; doi:10.1002/acn3.51526)
Supplement: Supplementary file 1 — File S1. Cluster Formula Utilized to Predict percent difference IsoTorque. For determination of percent difference IsoTorque, a Self‐Operating Map cluster analysis resulted in three clusters and an optimal Cubic Cluster Criterion of −31.201. The resultant principle components (eigenvalues of 1.61, 1.00, and 0.39) were utilized in a standard least squares regression to predict percent difference IsoTorque. The model (R 2 = 0.33; p < 0.001) demonstrated excellent differentiation of isoinertial torque between groups with MS different from OM and CTRL for all three clusters (p < 0.001) and OM different from CTRL for clusters 2 and 3 (p < 0.001). [file ACN3-9-515-s001.docx]

**Supplementary File**

Cluster Formula:

IfMin(

((:Name( "Avg FR (pps)" ) - 12.6078600943392) / 4.01380900581432) ^ 2 + ((

:DeltaF - (-1.65067491899865)) / 3.37687388110703) ^ 2 + ((

:Name( "Log [MUAP Peak2Peak Amp]" ) - 5.21062797706781) / 0.623027710258623) ^ 2,

**1**,

((:Name( "Avg FR (pps)" ) - 16.7804056477131) / 4.01380900581432) ^ 2 + ((

:DeltaF - 1.48574047734984) / 3.37687388110703) ^ 2 + ((

:Name( "Log [MUAP Peak2Peak Amp]" ) - 4.46599277012744) / 0.623027710258623) ^ 2,

**2**,

((:Name( "Avg FR (pps)" ) - 19.8762649832022) / 4.01380900581432) ^ 2 + ((

:DeltaF - (-2.80273183716201)) / 3.37687388110703) ^ 2 + ((

:Name( "Log [MUAP Peak2Peak Amp]" ) - 4.06824032033541) / 0.623027710258623) ^ 2,

**3**

)
